# Assessing the risk of cardiovascular events in patients receiving immune checkpoint inhibitors

**DOI:** 10.3389/fcvm.2022.1062858

**Published:** 2022-12-01

**Authors:** María Torrente, Mariola Blanco, Fabio Franco, Yago Garitaonaindia, Virginia Calvo, Ana Collazo-Lorduy, Lourdes Gutiérrez, Juan Cristóbal Sánchez, Aranzazu González-del-Alba, Roberto Hernández, Miriam Méndez, Blanca Cantos, Beatriz Núñez, Pedro A. C. Sousa, Mariano Provencio

**Affiliations:** ^1^Department of Medical Oncology, Puerta de Hierro Majadahonda University Hospital, Madrid, Spain; ^2^Faculty of Health Sciences, Francisco de Vitoria University, Madrid, Spain; ^3^Department of Electrical Engineering, NOVA School of Science and Technology, Universidade Nova de Lisboa, Lisbon, Portugal

**Keywords:** cardiovascular event, immune checkpoint inhibitors, myocarditis, cardiotoxicity, risk factors

## Abstract

**Background:**

Immune checkpoint inhibitors (ICIs) have revolutionized cancer treatment. However, despite their excellent therapeutic effect, these medications typically result in a broad spectrum of toxicity reactions. Immune-related cardiotoxicity is uncommon but can be potentially fatal, and its true incidence is underestimated in clinical trials. The aim of this study is to assess the incidence and identify risk factors for developing a cardiac event in patients treated with ICIs.

**Methods:**

We conducted a single-institution retrospective study, including patients treated with ICIs in our center. The main outcomes were cardiac events (CE) and cardiovascular death.

**Results:**

A total of 378 patients were analyzed. The incidence of CE was 16.7%, during a median follow-up of 50.5 months. The multivariable analysis showed that age, a history of arrhythmia or ischemic heart disease, and prior immune-related adverse events were significantly associated with CE.

**Conclusion:**

CE during ICI treatment are more common than currently appreciated. A complete initial cardiovascular evaluation is recommended, especially in high-risk patients, being necessary a multidisciplinary approach of a specialized cardio-oncology team.

## Introduction

The hallmark of many cancers is their ability to avoid the host immune response, allowing cell proliferation and metastasis (1). Cytotoxic T lymphocyte-associated antigen-4 (CTLA-4) and programmed cell death protein (PD-1) are co-inhibitory T-cell surface molecules, which downregulate the immune response by attenuating T-cell activation, proliferation, and cytokine production (2, 3). The immune system employs these inhibitory pathways to maintain T-cell tolerance and prevent autoimmunity (4). Numerous cancer cells overexpress the programmed cell death ligand 1 (PD-L1) on their surface, which contributes to their immune evasion by enhancing their immune escape ability, resulting in a poor prognosis for the patient (5). Based on these inhibitory molecules, several monoclonal antibodies targeting these immune checkpoint pathways have been developed in the last decade.

Immune checkpoint inhibitors (ICIs) have revolutionized the therapeutic landscape of many hematological and solid tumors. Since the introduction of the first ICI, ipilimumab (human IgG1 k anti-CTLA-4 monoclonal antibody) in 2011, six additional ICIs have been approved for cancer therapy by regulatory agencies (6), becoming a mainstay in the treatment of several neoplasms, including non-small cell lung cancer (NSCLC) and malignant melanoma. ICIs have shown high and durable response rates, either alone or in combination with other therapies, improving the survival of patients with advanced-stage malignancies, historically endowed with a poor prognosis (7, 8).

However, these agents also produce a wide spectrum of immune-related adverse events (irAEs), mainly due to aberrant autoreactive T-cell activation (9). These immune-mediated toxicities may affect any organ or tissue, with the most frequently reported being the skin, gastrointestinal system, and endocrine system (10). The incidence of irAEs varies between CTLA-4 inhibitors and PD-1 inhibitors, being high-grade adverse events more common with the combination ICI therapy (11).

Immune-related cardiotoxicity is a potentially fatal irAE. Since the first specific case of ICI-associated cardiotoxicity was reported in 2014 (12), cardiotoxicity during ICI treatment has been increasingly reported (13–15). The current evidence available arises from pharmacovigilance databases, case reports, and retrospective series (16), but no randomized controlled trials have assessed the potential cardiotoxic role of ICIs. Among the various forms of ICI cardiotoxicities, myocarditis is the most frequently reported due to its high morbidity and mortality. Retrospective evaluation literature has estimated that the incidence of ICI-related myocarditis ranges from 0.09 to 1.14% (17, 18). Nevertheless, the true incidence of other cardiovascular effects of immunotherapy is uncertain. Immune-related cardiotoxicity involves almost all parts of the heart, being the myocardium the most sensitive to ICIs toxicity. The potential mechanism of ICI-mediated cardiac toxicity is not fully understood, but histological analysis has revealed that the infiltration of CD4+/CD8+ T-cells and macrophages in the heart tissue may play a key role in the pathogenesis (19). ICI-related cardiotoxicity can be inflammatory, including conditions such as pericarditis, myocarditis, and perimyocarditis, or non-inflammatory, including left ventricular dysfunction without myocarditis, Takotsubo-like syndrome, coronary vasospasm, arrhythmias, and myocardial infarction (20). Various reports have revealed that immune-related cardiotoxicity may occur early after exposure to an ICI, suggesting a potential predisposition to cardiotoxicity that is possibly associated with pre-existing cardiovascular risk factors (CVRF) (21). Likewise, as reported with other irAEs, a combined ICI therapy with anti-CTLA4 and anti-PD-1/PD-L1, significantly increases the risk of developing an immune-related CE (22), as well as previous administration of other cardiotoxic cancer therapies, such as anthracyclines, trastuzumab, thoracic radiotherapy, or antiangiogenic therapy.

In order to gain a more robust understanding of immune-related cardiotoxicity, we reviewed the incidence of CE developed during ICI treatment in our institution. In addition, we tried to assess the role of CVRF and other cancer therapies in the development of cardiac toxicity.

## Materials and methods

### Study population

We conducted a single-institution retrospective study at the Puerta de Hierro-Majadahonda University Hospital in Madrid, Spain. Oncologic patients treated in our center with immune checkpoint inhibitors, either in monotherapy or in combination with chemotherapy or other therapies, were included from March 2014 to November 2020.

Clinical history from electronic health records was retrieved, and specific data were collected regarding demographics, previous CVRF, and oncological features such as specific tumor, histology, AJCC/UICC stage, treatments received (including type of chemotherapy and targeted therapies, with an emphasis on those with cardiotoxic effects), treatment-related toxicity, and outcomes (overall survival).

Cardiac events identified during or after immune checkpoint inhibition were electrocardiographic alterations (such as long QT syndrome, any grade bundle branch or atrioventricular blockades or atrial fibrillation), congestive heart failure, pulmonary embolism, acute coronary syndrome, pericarditis, and myocarditis. Treatment interruptions, hospitalizations due to cardiologic issues, and cardiac-related deaths were also identified.

The aim of the study was to determine the incidence of cardiac events in patients exposed to immune checkpoint inhibitors and to identify clinical factors for predicting the onset of these events.

### Statistical analysis

Qualitative variables are expressed as an absolute value and percentage. Quantitative variables are expressed as the median and interquartile range (IQR). To test for clinical data categorical associations with cardiac event development, we performed a Chi-square test in variables such as sex, known CVRF (hypertension, diabetes, valve disease, chronic kidney disease, ischemic heart disease, previous history of arrhythmia, smoking habit, tumor location, previous potentially cardiotoxic drug exposure, previous chest radiotherapy, and tumor objective response). T-student test was used for age. To identify risk factors increasing the probability of CE, Cox models were constructed. Risk factors with a *P*-value lower than 0.05 in the univariable analysis were developed into multivariable models. Odds ratios (ORs) and 95% confidence intervals (CIs) were provided for the multivariable model. In line with recommendations, the cumulative incidence was designed up to the 95 percentile of follow-up duration.

Based on the results obtained in the multivariable analysis, as well as clinically important variables, we developed a predictive score for the risk of developing a cardiac event after immunotherapy exposure. The score calibration was assessed using the Hosmer–Lemeshow goodness-of-fit test as well as the discrimination through the area under the receiver operating characteristic curve.

All statistical analyses were performed using Stata version 23 software.

### Ethics

Ethical approval was granted by the Puerta de Hierro-Majadahonda University Hospital Clinical Research Ethics Committee. The study was carried out in accordance with the requirements expressed in the Declaration of Helsinki, as well as with the current legislation in Spain on conducting observational studies (Ministerial Order SAS/3470/2009).

## Results

### Baseline characteristics of the full cohort

Between March 2014 and November 2020, a total of 378 patients receiving ICIs treatment in our institution were analyzed. Of those, 134 were females (35.5%) and 244 were males (64.6%). The median age was 61 years old (p25–p75: 55–68).

Table 1 lists the main baseline characteristics of our cohort of patients. The most frequent comorbidities were hypertension (40.7%), dyslipidemia (30.4%), and type 2 diabetes (13.2%); 29.9% of patients were current smokers at the time of the analysis, and 51.3% were former smokers. Regarding other more specific CVRF, the most prevalent was peripheral vascular disease (17.2%), followed by history of coronary heart disease (5.0%), heart valve disease (5.0%), chronic renal failure (4.5%), and congestive HF (0.5%); 24 patients (6.4%) had history of arrhythmia being the most frequent atrial fibrillation (83.4%). In addition, 10.9% of the patients were receiving anticoagulants and 11.9% antiplatelet treatment. Only 41 (10.8%) patients had a family history of cardiovascular disease. In the cardiologic evaluation prior to the start of ICI treatment, 20 patients (5.3%) presented with alterations in cardiac contractility, and 25 (6.6%) had left ventricular hypertrophy in the echocardiographic assessment. Only 2 patients had evaluable cardiomegaly in chest X-ray.

**TABLE 1:** Baseline characteristics of patients at the time of initial administration of immune checkpoint inhibitors.

Variable	*N* = 378 patients (%)
Sex	Male: 244 (64.6%) Female: 134 (35.5%)
Age (median)	61.0 (p25–p75: 55–68)
Hypertension	154 (40.7%)
Diabetes	50 (13.2%)
Hypercholesterolemia	115 (30.4%)
Current or prior smoking	307 (81.2%)
Coronary syndrome	19 (5.0%)
Heart failure	2 (0.5%)
Chronic renal failure	17 (4.5%)
Valvular disease	19 (5.0%)
Arrhythmia	24 (6.4%)
Peripheral arterial disease	65 (17.2%)
Type of tumor	Lung cancer: 236 (62.3%) Melanoma: 48 (12.7%) Renal cell carcinoma: 19 (5.0%) Bladder cancer: 18 (4.8%) Head and neck tumors: 16 (4.2%) Hepatocellular carcinoma: 9 (2.4%) Lymphoma: 8 (2.1%) Colorectal cancer: 5 (1.3%) Gastric cancer: 5 (1.3%) Gynecological tumors: 3 (0.8%) Breast cancer: 3 (0.8%) Thymoma: 2 (0.5%) Cholangiocarcinoma: 1 (0.3%) Esophagus tumors: 1 (0.3%)
Tumor stage when ICI was started	I: 4 (1.0%) II: 8 (2.1%) III: 52 (14.0%) IV: 313 (82.8%)
Previous cardiotoxic treatments	Anthracyclines: 13 (3.4%) Cisplatin: 87 (23.0%) Antimicrotubule: 110 (29.1%) Antiangiogenic therapy: 49 (13.0%) HER2 targeted therapy: 2 (0.5%) Thoracic radiotherapy: 95 (25.1%)

ICI, immune checkpoint inhibitors.

Regarding tumor characteristics, most of the patients receiving ICI treatment were lung cancer patients (62.3%), followed by melanoma (12.7%), renal cell carcinoma (5.0%), urothelial carcinoma (4.8%), and head-and-neck tumors (4.2%). The predominant histology was adenocarcinoma (42.9%) followed by squamous cell carcinoma (20.6%) and melanoma (12.7%). Most of the patients were at stage IV of the disease (82.2%). 34.4% of patients received ICI in the first-line setting, in monotherapy, or in combination with other therapies. Regarding the type of ICI treatment administered, most of the patients received anti-PD1 drugs (64.8%), followed by anti-PDL1 drugs (9%) and anti-CTLA4 drugs (1.6% in monotherapy and 9.5% in combination with anti-PD1), with a median of 6 cycles per patient. Details on ICI treatment are listed in Table 2. When we evaluated the best response to ICIs by radiologic assessment, 32.3% of patients had a partial response, 13% stable disease, and 9.2% complete response. Nevertheless, most of the patients had progressive disease at first evaluation (43.1%). At the time of the analysis, 335 patients (88.6%) had interrupted the ICI therapy, most of them due to disease progression (51.6%) or death (10.6%), end of treatment after 2 years completion (14.2%) and only 37 (9.8%) due to immune-related toxicity. The most frequent irAE was gastrointestinal (18.9%), followed by skin disorders (16.5%) and pneumonitis (16.5%). 45 patients experienced grade 3 adverse events (11.9%). Of note, only one patient interrupted ICI treatment due to SARS-CoV-2 infection.

**TABLE 2:** Type of ICI treatment received.

ICI-treatment	
° ICI-monotherapy	Anti-PD1: 245 (64.8%) Anti-PDL1: 34 (9.0%) Anti-CTLA4: 6 (1.6%)
° ICI-combinations	Anti-CTLA4 + anti-PD1: 36 (9.5%) ICI + chemotherapy: 49 (13.0%) ICI + VEGF inhibitors: 7 (1.8%) ICI + tyrosine kinase inhibitors: 1 (0.3%)

ICI, immune checkpoint inhibitors; VEGF, vascular endothelial growth factor.

As abovementioned, most patients presented advance stage of the disease upon ICIs initiation, and had progressed to several lines of treatment, including potentially cardiotoxic drugs. Specifically, 3.4% of patients had previously received anthracyclines, 23.0% cisplatin, 29.1% antimicrotubule agents, 13% antiangiogenic therapy, and only 0.5% (2 patients) anti-HER2 therapy. Regarding radiotherapy, 95 patients (25.1%) had received thoracic radiotherapy (including lung/mediastinum in 80%), most of them with radical/curative intention (median dose of 60 Gy). Only 54 patients received concomitant chemotherapy (55% platinum-based chemotherapy).

### Incidence of cardiac events in the full cohort

**FIGURE 1:**
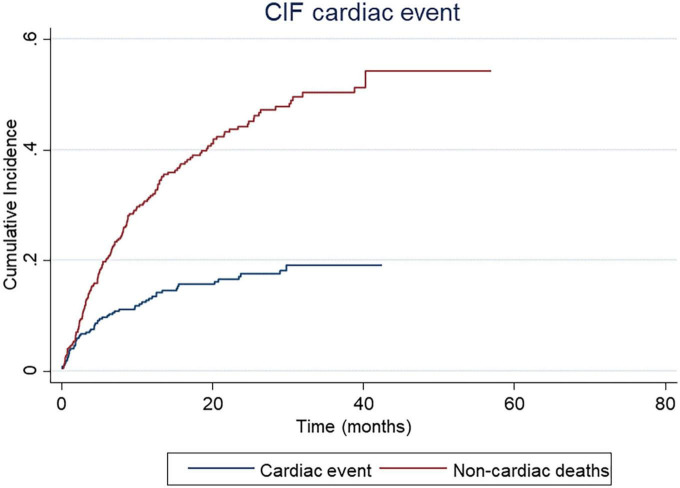
Cumulative Incidence Function (CIF) of cardiac events.

Focusing on the CE, 63 patients (16.7%) presented a CE during ICI treatment in our study (with some patients presenting more than one CE). The median time to CE onset from the initiation of ICI therapy was 4.3 months (p25: 1.5–p75: 11.1). Table 3 summarizes the incidence of each CE. Considering non-cardiac death as a competing event, the cumulative incidence rates for a CE were 10.2% at 6 months, 13.8% at 12 months, 16% at 18 months, 17.7% at 24 months, 18.8% at 6 months, and 19.8% at 48 months (Figure 1). The most prevalent event was an electrocardiographic alteration (69.8% of all CE), being atrial fibrillation the most frequently reported. Among the 44 patients who developed some type of arrhythmia during immunotherapy, 42 were newly onset and only 4 had history of atrial fibrillation. Congestive HF accounted for 19.0% of all CE; of important note, we found that all of these patients had previous CVRF, which could have predisposed to the subsequent HF event during ICIs. However, none of these patients had a history of HF or echocardiographic abnormalities prior to ICI therapy. Pulmonary embolism represented 10.8% of all CE, having all of them predisposing factors such as recent surgery, immobilization, or disease progression. None required fibrinolysis or ICU admission, and 3 patients (4.6% of all CE) developed an ACS, of whom, two had a previous history of coronary heart disease and were under cardiologic follow-up.

**TABLE 3:** Global incidence of cardiovascular events during ICI treatment.

Cardiac event	Incidence
Electrocardiographic alterations	12.2%
Pulmonary embolism	1.9%
Mace	
• Heart failure	3.4%
• Acute coronary syndrome	0.8%
Myocarditis	0.5%
Pericardial disease	0.5%
Mortality	
• Global mortality	53.4%
• Cardiovascular mortality	0.8%

MACE, major adverse cardiovascular events.

Regarding the “inflammatory” cardiotoxicity, two patients were diagnosed with pericarditis and two with myocarditis. These patients required hospital admission and close cardiologic monitoring, as well as high-dose corticosteroids, and, in the case of myocarditis, immunosuppressive treatment. Three of these four patients diagnosed with peri- or myocarditis presented other concomitant irAE, such as myasthenia gravis, polyarthritis, or Guillain–Barre syndrome. As previously stated, 37 patients (9.8%) had to interrupt treatment because of an irAE, but only 2 patients (0.5%) due to specific cardiac toxicity. During a median follow-up of 50.5 months (95% CI 45.5–57.7), 202 patients (53.4%) died, of whom 3 (0.8%) the cause of death was due to a CE: 2 fatal events associated to myocarditis and 1 to congestive HF.

The cumulative incidence function (CIF) represents the incidence rates of cardiac events in our cohort of patients, considering non-cardiac deaths as a competing event. Probably, the incidence of CE might be underestimated due to the high mortality rates observed in cancer patients.

### Risk factors for cardiac events

Univariable and multivariable Cox regression analyses are presented in Table 4. Older patients and those with history of heart valve disease, ischemic heart disease, or arrhythmia were prone to develop a CE in the univariable analysis. However, only age (OR 1.03; 95% CI 0.99–1.06; *p* = 0.05), a history of ischemic heart disease (OR 3.25; 95% CI 1.16–9.14; *p* = 0.025), or arrhythmia (OR 4.11; 95% CI 1.66–10.22; *p* = 0.002), remained significantly associated with CE in the multivariable analysis. Likewise, those patients who had developed another irAE (31.7%) were also at increased risk (OR 2.08; 95% CI 1.11–3.94; *p* = 0.023). By contrast, other known CVRF such as hypertension, diabetes, dyslipidemia, chronic kidney failure, or smoking habit, did not increase the risk for cardiac toxicity in our study, nor did the type of tumor, stage, or response to treatment. Regarding ICI therapy, there were no differences between combination regimens (with other ICI, antiangiogenic therapy, or chemotherapy) and monotherapy (*p* = 0.87). Similarly, patients who had received other cardiotoxic therapies (anthracyclines, cisplatin, antimicrotubule agents, antiangiogenic therapy, anti-HER2 therapy, or thoracic radiotherapy), were not at increased risk for cardiac toxicity.

**TABLE 4:** Univariable and multivariable Cox regression analysis.

	No cardiac event (*n* = 313)	Cardiac event (*n* = 65)	Univariable analysis, *P*-value	Multivariable analysis OR (95% CI), *P*-value
Sex				
• Male	204 (65.2%)	40 (61.5%)	0.557	
• Female	109 (34.8%)	25 (38.5%)		
Clinical factors:				
• Age	59.68 (58.4–61.0)	64.82 (62.5–67.1)	0.001*	**OR 1.03, 95% CI 0.99–1.06** *p* = 0.05*
• Hypertension	125 (39.9%)	29 (44.6%)	0.485	
• Dyslipidemia	89 (28.4%)	26 (40.0%)	0.065	
• Diabetes mellitus	40 (12.8%)	10 (15.4%)	0.074	
• Valve heart disease	11 (3.5%)	8 (12.3%)	0.003*	OR 1.51, 95% CI 0.48–4.76 *p* = 0.480
• Chronic kidney disease	13 (4.2%)	4 (6.2%)	0.479	
• Ischemic heart disease	12 (3.8%)	7 (10.8%)	0.020*	**OR 3.25, 95% CI 1.16–9.14** *p* = 0.025*
• Arrhythmia	13 (4.2%)	11 (16.9%)	0.000*	**OR 4.11, 95% CI 1.66–10.22** *p* = 0.002*
Type of tumor			0.060	
Stage			0.394	
Previous therapies				
• Anthracycline	10 (3.2%)	3 (4.6%)	0.567	
• Cisplatin	74 (23.6%)	13 (20.0%)	0.526	
• Antimicrotubule	92 (29.4%)	18 (27.7%)	0.784	
• Antiangiogenic	43 (13.7%)	6 (9.2%)	0.325	
• AntiHer2	1 (0.3%)	1 (1.5%)	0.218	
• Thoracic radiotherapy	74 (24.0%)	20 (30.8%)	0.250	
Best response			0.345	
Other irAE	58 (18.5%)	20 (30.8%)	0.027*	**OR 1.08, 95% CI 1.11–3.94** *p* = 0.023*

ORs and 95% CIs were derived from adjusted Cox proportional hazards model. irAEs, immune-related adverse events; OR, odds ratio; CI, confidence interval. *Statistically significant (*p* < 0.05). Bold values represent the statistically significant results (*p* < 0.05).

## Discussion

This study provides real-world observational data on the risk of CE associated with ICI treatment, which can extend beyond myocarditis. Their detection is currently challenging due to the lack of consistency of its clinical manifestation, and, consequently, their incidence is probably underestimated in clinical trials. Thus, prospective or retrospective studies with larger cohorts are essential for capturing the true incidence of CE in daily practice. A pharmacovigilance study using VigiBase, the WHO’s global database of individual case safety reports, reported significantly higher, but still very low incidences of myocarditis and pericarditis, with an incidence of 0.39 and 0.30%, respectively (23). A Danish nationwide study evaluated the risk of CE in ICI-treated patients, reporting an absolute risk at 1 year of 9.7% in 743 patients with lung cancer and 6.6% in 145 patients with melanoma. They concluded that the hazard rates of CE were higher in patients with vs. without ICI treatment (24).

The most prevalent CE reported in our study were arrhythmias, of which, atrial fibrillation was the most prevalent one. ICI-mediated arrhythmias and conduction diseases, in the absence of generalized myocarditis, are emerging as a more frequent and potentially serious cause of ICI-mediated sudden death (21). The mechanisms underlying the arrhythmias are unknown, but might include inflammation of the His–Purkinje conduction system, increased systemic inflammatory state, myocarditis with inflammation and fibrosis, and other causes of arrhythmias in cancer patients (QT-interval prolonging drugs, electrolyte imbalances, myocardial metastases) (20). Other relevant CE reported in our study were HF and ACS, which can be considered MACE. Although other well-known CVRF, such as hypertension, diabetes, or dyslipidemia, are major risk factors for developing a MACE, and immunotherapy seems to play an important role in the pathogenesis of these events. Preclinical models have shown that cardiomyocyte PD-L1 expression is upregulated in cardiac stress, including ischemia reperfusion and left ventricular hypertrophy, and might have cardioprotective actions by suppressing excessive myocardial inflammation (25). Likewise, it is known that atherosclerosis is characterized by low-grade chronic inflammation, and that acute or chronic inflammatory conditions can accelerate plaque rupture (26). Thus, ICIs could induce acceleration or decompensation of pre-existing HF and ACS in susceptible individuals. In line with this, in a matched cohort study of 5,684 patients, a threefold higher risk of atherosclerotic CE (myocardial infarction, coronary revascularization, and ischemic stroke) was observed after starting ICI therapy (27). Additionally, HF is not a marginal issue in this study and requires a better understanding of the systemic perturbation induced by immune checkpoints and its impact on cardiac function. Patients with dilated cardiomyopathy can have heterogeneous etiology such as genetic, viral, immunological, and environmental factors. Despite several animal studies that indicate that PD-1 may be an important factor contributing to the prevention of autoimmune diseases (28, 29), the involvement of an immune mechanism in patients with dilated cardiomyopathy is still controversial.

Regarding pulmonary embolisms, the evidence is scarce. Some reports suggest that these events may be associated with ICI therapy (30); however, this should be viewed as speculative and requires further investigation. In our cohort, the incidence of myocarditis and pericardial diseases was 0.5. Our rate is similar to that previously reported in other studies, although there has been a substantial increase in reporting incidence over time (31), probably due to an increased use of ICIs, along with heightened recognition of this new clinical entity. With a fatality rate of 27–46%, ICI- related myocarditis is the most lethal form of irAE (32), requiring high-dose corticosteroids and, in most cases, immunosuppressive treatment. The pathophysiology of ICI-induced myocarditis is incompletely understood. Post-mortem evaluations have observed inflammatory T-cell (CD4+ and CD8+) and macrophage infiltrate, as well as loss of cardiomyocytes, which, as mentioned before, might also employ the PD-1/PD-L1 pathway to prevent T-cell hyperactivation in the heart in physiological conditions (17). These findings are consistent with the mechanism of ICI action and can be considered an on-target toxicity.

The presence of pre-existent CVRF was common in our study population (46.6%), similar to that reported in the literature (33). Little is known about predisposing risk factors for ICI-related cardiotoxicity. Mahmood et al. reported that diabetes mellitus may predispose to immune-related myocarditis, but no such association has yet been found for other CVRF (18). In a recent retrospective match-cohort study of 672 patients treated with ICIs, only age, history of HF, and heart valve disease were independently associated with MACE (34). In our study, the multivariable analysis showed that older patients, those with history of ischemic heart disease or arrhythmias, and those who developed an irAE are at increased risk of developing a CE during ICI treatment. Other non-cardiac irAEs are frequent, occurring in approximately half of the patients with ICI-mediated myocarditis (18). Myositis is one of the most prevalent (23–25%) (21, 31), and this association might reflect a shared antigen profile and immune phenotype between cardiac and skeletal muscle.

A combination of ICI therapy seems to be a clear risk factor for developing a CE according to the literature. Johnson et al. reported a higher incidence (0.27 vs. 0.06%) and severity (60 vs. 10%) of myocarditis in patients receiving a combination of nivolumab and ipilimumab compared to nivolumab alone (17). Similarly, combined immunotherapy and other cardiotoxic cancer therapy (such as anti-VEGF-tyrosine kinase inhibitors, platinum-based chemotherapy, or radiotherapy) seem to increase the vulnerability for developing a CE (35). However, our study was unable to demonstrate those associations; possibly, a larger study sample might have exposed a statistical difference. On the other hand, many patients with metastatic disease have received multiple treatments before initiating ICIs treatment, some of which might have been cardiotoxic. By inducing myocardium damage, these treatments can lead to the exposure of cardiac antigens developing immune responses that can be amplified upon the initiation of ICIs (36). In our cohort, previous treatment with cardiotoxic therapy was common (58.9%), but no association was observed with the development of CE in the multivariable analysis.

**FIGURE 2:**
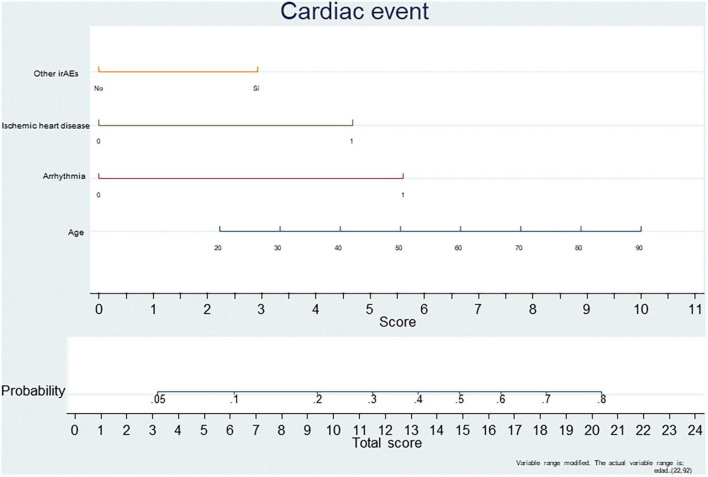
Predictive risk score for developing a cardiac event during ICI treatment.

Our real-world data support a baseline and protocoled cardio-oncology follow-up of high-risk patients, such as elderly patients and those with pre-existing cardiovascular comorbidities, especially history of ischemic heart disease and arrhythmias. In addition, patients who develop another irAE should be included in this high-risk group. Based on these results, we have designed a predictive risk-score, which could potentially assess the probability of developing a CE based on the presence or absence of these specific risk factors (Figure 2). This predictive risk score is based on four different variables associated with the development of a CE in our study. Depending on the presence or absence of each variable, the patient gets a score which might predict the probability of developing a cardiac event.

No evidence-based algorithm yet exists for the management of these patients, mainly due to the absence of prospective trials. Two risk scores in the setting of breast cancer, specifically in early-stage HER2-positive breast cancer patients treated with trastuzumab have been reported. In 2012, Romond et al. used 7-year follow-up data from the National Surgical Adjuvant Breast and Bowel Project B-31 adjuvant trastuzumab study to derive a prediction tool for cardiotoxicity (37). A second prediction tool was derived by Ezaz et al., who used data from the U.S. Surveillance, Epidemiology, and End Results database in 1,664 women with previous exposure to trastuzumab (38). Moreover, the CHEMO−RADIAT study validated a predictive model for risk of major cardiovascular events after the diagnosis of breast cancer based on both conventional cardiovascular risk factors and breast cancer treatment−related cardiovascular risk factors, showing a good performance but only valid for breast cancer and again, based only on CVRF. The main limitation of these cardiotoxicity scores is that they focus on CVRF but do not incorporate damage cardiac biomarkers such as BNP or troponins, nor novel echocardiography parameters such as global longitudinal strain (GLS), used in clinical practice for early detection of changes in myocardial contractile function (39, 40).

In this sense, our score has two advantages: one, it is valid for any type of cancer, and two, that it includes cardiac biomarkers determination and echocardiography exams. Nevertheless, it needs to be validated in the different pathologies in a larger sample. Thus, a thorough cardiovascular evaluation is essential before starting ICI treatment, necessary for the multidisciplinary assessment of a cardio-oncology team.

The American Society of Clinical Oncology (ASCO) recommends a baseline cardiac workup before initiating potential cardiotoxic therapy, and, in case of symptoms, an extended cardiology workup including echocardiography, chest x-ray, and cardiac biomarkers, such as cardiac troponin and brain natriuretic peptide (BNP) (41). On the other hand, other studies have also suggested a surveillance strategy for high-risk patients who are receiving ICI treatment (20). The baseline cardiac assessment pre-ICI should include a complete clinical history and risk factor assessment, electrocardiogram (ECG), cardiac troponin, BNP, and echocardiogram. In high-risk patients, a closer monitoring is recommended, with ECG and cardiac biomarkers determination before ICI initiation and dose-adjustment. These specific tests are proposed because ICI-related CE has been characterized consistently by BNP elevation or conduction disease. Measurement of cardiac troponin should also be considered because it is more specific for myocarditis, the most important ICI-triggered cardiotoxic effect. Given the heterogeneity in the risk of cardiotoxic effects, a personalized strategy based on the patient´s baseline risk assessment may be relevant. Upon cardiotoxicity suspicion, it is recommended to immediately interrupt ICI therapy, repeat all cardiovascular exams, and refer the patient to a cardio-oncology specialist (20). In case of major cardiovascular complications, management should be in accordance with the current European Society of Cardiology (ESC) and American Heart Association (AHA) guidelines.

The main limitation of this study is its retrospective, descriptive nature, based on the activity conducted in a single center, Puerta de Hierro-Majadahonda University Hospital. Consequently, the incidence of CE might have been underestimated due to the lack of information in the medical records.

## Conclusion

The real incidence of immune-related cardiotoxicity in real-world practice is higher than that reported in clinical trials. Cardiovascular history, older age, and prior irAEs are potential risk-factors, making patients more susceptible to these events. A thorough cardiovascular evaluation is strongly recommended before ICI treatment and, in high-risk patients, a closer follow-up and prompt referral to a cardio-oncology specialist as soon as cardiotoxicity is suspected. Risk stratification using a risk scoring system may improve the cardiovascular outcomes of patients treated with ICIs by identifying the patients at risk for cardiovascular events. Incorporation of clinical biomarkers and imaging parameters should be incorporated into these scores in order to improve the accuracy of prediction. Future studies with larger samples are needed in order to validate risk scores and develop standardized follow-up strategies for these patients.

## Data availability statement

The raw data supporting the conclusions of this article will be made available by the authors upon reasonable request.

## Ethics statement

The studies involving human participants were reviewed and approved by the Comité de Ética de Investigación con Medicamentos del Hospital Universitario Puerta de Hierro Majadahonda. The patients/participants provided their written informed consent to participate in this study.

## Author contributions

MT, MB, FF, and MP drafted and designed the study. MM, BC, LG, MB, RH, AC-L, VC, JS, AG-d-A, and BN performed patients’ inclusion. MT, MB, PS, and MP drafted the manuscript. All authors reviewed and approved the manuscript and read and agreed to the published version of the manuscript.
